# Correlation between *MTHFR* polymorphisms and glaucoma: A meta‐analysis

**DOI:** 10.1002/mgg3.538

**Published:** 2019-03-09

**Authors:** Ling Zhang, Bin Chen

**Affiliations:** ^1^ Department of Ophthalmology People’s Hospital of Leshan Leshan China

**Keywords:** gene polymorphisms, glaucoma, meta‐analysis, methylenetetrahydrofolate reductase (MTHFR)

## Abstract

**Background:**

Whether methylenetetrahydrofolate reductase (*MTHFR*) polymorphisms are implicated in glaucoma remains controversial. Therefore, we performed this study to better assess the relationship between *MTHFR* polymorphisms and the likelihood of glaucoma.

**Methods:**

A systematic research of PubMed, Medline, and Embase was performed to retrieve relevant articles. Odds ratios (ORs) and 95% confidence intervals (CIs) were calculated.

**Results:**

A total of 18 studies with 7,168 participants were analyzed. In overall analyses, a significant association with the likelihood of glaucoma was detected for the rs1801133 polymorphism in dominant (*p* = 0.04, OR = 0.90, 95%CI 0.81–1.00) and allele (*p* = 0.02, OR = 0.91, 95%CI 0.84–0.98) comparisons. Further, subgroup analyses by ethnicity revealed that both rs1801131 and rs1801133 polymorphisms were significantly associated with the likelihood of glaucoma in West Asians. However, no positive results were detected for two investigated polymorphisms in East Asians and Caucasians.

**Conclusion:**

Our findings indicated that rs1801131 and rs1801133 polymorphisms may serve as genetic biomarkers of glaucoma in West Asians.

## 1 INTRODUCTION

Glaucoma, characterized by atrophy of optic nerve and progressive loss of sight, is one of the leading causes of blindness all over the world (Mantravadi & Vadhar, 2015). Over the past two decades, the prevalence of glaucoma has significantly increased, and according to an epidemiological study, it is estimated that glaucoma would affect over 80 million people by 2020 (Weinreb, Aung, & Medeiros, 2014). So far, the exact cause of glaucoma is still largely unclear. Nevertheless, the fact that multiple genetic loci have found to be correlated with individual susceptibility to this disease suggested that genetic factors play crucial roles in the occurrence and development of glaucoma (Asefa, Neustaeter, Jansonius, & Snieder, 2018; Cissé, Bai, & Meng, 2018; Wiggs & Pasquale, 2017).

Methylenetetrahydrofolate reductase (MTHFR) is a central regulator of folate metabolism and homocysteine synthesis (Trimmer, 2013). Previous studies have demonstrated that MTHFR deficiency could lead to hyperhomocysteinemia and give rise to the development of multiple vascular and neurodegenerative disorders including glaucoma (Li, Xu, Zeng, Gong, & Lan, 2016; Zacharaki et al., 2014). As a result, functional *MTHFR* (NG_013351.1) polymorphisms are thought to be ideal candidate genetic biomarkers of glaucoma.

Until now, some pilot studies have investigated possible correlations of *MTHFR* rs1801131 (A1298C) and rs1801133 (C677T) polymorphisms with glaucoma, but the results of these studies were conflicting and the sample size of individual studies was relatively small (Al‐Shahrani et al., 2016; Buentello‐Volante et al., 2013; Gupta et al., 2014; Nilforoushan et al., 2012). Therefore, we performed the present meta‐analysis to better elucidate the relation between *MTHFR* polymorphisms and glaucoma.

## 2 MATERIALS AND METHODS

### 2.1 Ethical compliance

This article does not contain any studies with human participants or animals performed by any of the authors, so ethical approval is not required.

### 2.2 Literature search and inclusion criteria

The current meta‐analysis was adhered to the Preferred Reporting Items for Systematic Reviews and Meta‐analyses (PRISMA) guideline (Moher, Liberati, Tetzlaff, Altman, & PRISMA group., 2009). PubMed, Medline, and Embase were searched for relative articles published before September 2018 using the following strategy: (*methylenetetrahydrofolate reductase* OR *MTHFR*) AND (polymorphism OR variant OR mutation OR genotype OR allele) AND (primary open‐angle glaucoma OR POAG OR primary closed angle glaucoma OR PCAG OR pseudoexfoliation glaucoma OR PXFG OR glaucoma). The reference lists of all retrieved publications were also manually screened to identify other potentially relevant articles.

To test the research hypothesis of this meta‐analysis, included studies should meet all the following criteria: (a) case–control study on correlation between *MTHFR* polymorphisms and the likelihood of glaucoma; (b) provide adequate data to calculate odds ratios (ORs) and 95% confidence intervals (CIs); (c) full text in English or Chinese available. Studies were excluded if one of the following criteria was fulfilled: (a) not relevant to *MTHFR* polymorphisms and glaucoma; (b) family‐based association studies; (c) case reports or case series; (d) abstracts, reviews, comments, letters, and conference presentations. For duplicate reports, we only included the study with the largest sample size for analyses.

### 2.3 Data extraction and quality assessment

The following data were extracted from all included studies: (a) name of first author; (b) year of publication; (c) country and ethnicity of participants; (d) the number of cases and controls; and (e) the genotypic distribution of *MTHFR* polymorphisms in cases and controls. Additionally, the probability value (*p* value) of Hardy–Weinberg equilibrium (HWE) was also calculated. The Newcastle‐Ottawa scale (NOS) was used to assess the quality of eligible studies (Stang, 2010). The NOS has a score range of zero to nine, and studies with a score of more than seven were thought to be of high quality. Two reviewers conducted data extraction and quality assessment independently. When necessary, the reviewers wrote to the corresponding authors for extra information or raw data. Any disagreement between two reviewers was solved by discussion until a consensus was reached.

### 2.4 Statistical analysis

All statistical analyses were conducted with Review Manager Version 5.3.3 (The Cochrane Collaboration, Software Update, Oxford, United Kingdom). ORs and 95% CIs were calculated to assess potential associations of *MTHFR* polymorphisms with the likelihood of glaucoma in all possible genetic models, and a *p* value of 0.05 or less was considered to be statistically significant. Between‐study heterogeneities were evaluated with *I* ^2^ statistic. If *I* ^2^ was greater than 50%, random‐effect models (REMs) would be used to pool the data in overall and subgroup analyses. Otherwise, fixed‐effect models (FEMs) would be employed for synthetic analyses. Sensitivity analyses were carried out to test the stability of our findings. Funnel plots were applied to evaluate possible publication bias.

## 3 RESULTS

### 3.1 Characteristics of included studies

**Figure 1:**
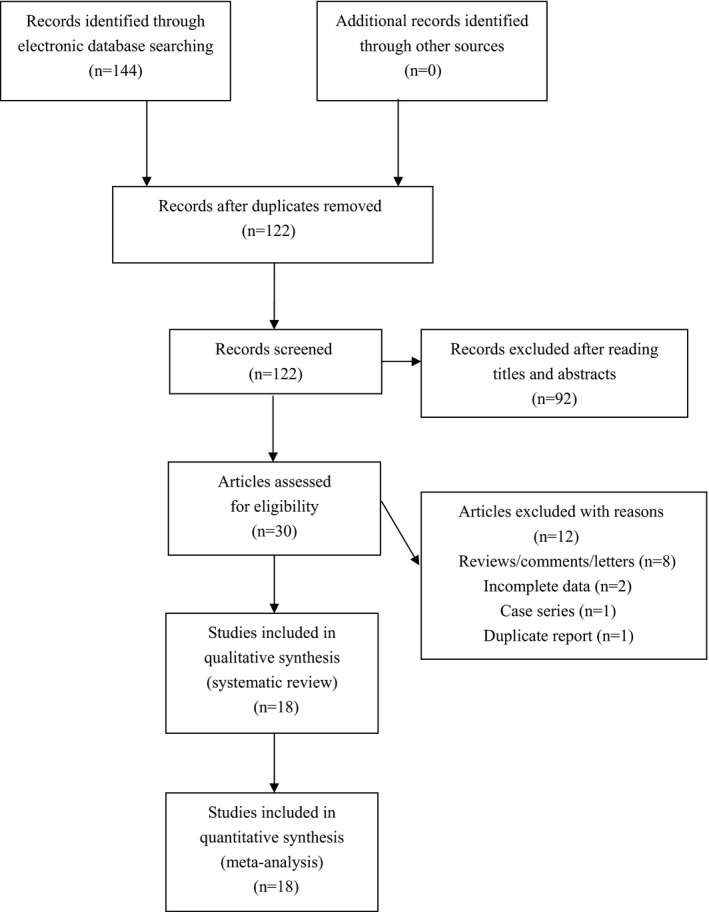
Flowchart of study selection for the present study

The literature search generated 144 results. After exclusion of irrelevant and duplicate articles by reading titles and abstracts, 30 articles were retrieved for further evaluation. Another 12 articles were subsequently excluded after reading the full text. Finally, a total of 18 eligible studies containing 3,453 cases and 3,715 controls were included for analyses (see Figure 1). Characteristics of included studies were summarized in Table 1.

**Table 1:** The characteristics of included studies

First author, year	Country	Ethnicity	Type of disease	Sample size	Genotype distribution		*p*‐value for HWE	NOS score
					Cases	Controls		
rs1801131								
Fan, 2008	USA	Mixed	PXFG	158/50	74/58/26	22/19/9	0.191	7
Mabuchi, 2006	Japan	East Asian	POAG	264/106	167/94/3	61/44/1	0.023	7
Micheal, 2009	Pakistan	West Asian	POAG	173/143	35/114/24	20/97/26	<0.001	7
Micheal, 2009	Pakistan	West Asian	PCAG	122/143	34/76/12	20/97/26	<0.001	7
Woo, 2009	Korea	East Asian	POAG	78/100	57/19/2	75/22/3	0.388	8
Zacharaki, 2014	Greece	Caucasian	POAG	66/133	33/27/6	64/56/13	0.883	8
Zacharaki, 2014	Greece	Caucasian	PXFG	74/133	31/32/11	64/56/13	0.883	8
Zetterberg, 2007	Sweden	Caucasian	POAG	243/187	119/97/27	88/87/12	0.117	7
rs1801133								
Al‐Shahrani, 2016	Saudi Arabia	West Asian	POAG	144/280	88/56/0	210/70/0	0.017	8
Al‐Shahrani, 2016	Saudi Arabia	West Asian	PCAG	66/280	49/17/0	210/70/0	0.017	8
Bleich, 2002	Germany	Caucasian	POAG	18/19	5/11/2	13/5/1	0.588	7
Buentello‐Volante, 2013	Mexico	Mixed	POAG	118/100	42/53/23	34/49/17	0.927	7
Clement, 2009	Australia	Caucasian	POAG	70/42	38/25/7	25/14/3	0.598	8
Clement, 2009	Australia	Caucasian	PXFG	48/42	18/23/7	25/14/3	0.598	8
Fan, 2008	USA	Mixed	PXFG	168/50	66/82/20	21/22/7	0.750	7
Fan, 2010	China	East Asian	POAG	397/201	244/137/16	135/60/6	0.830	7
Fingert, 2006	USA	Mixed	POAG	178/166	72/77/29	75/73/18	0.970	7
Fingert, 2006	USA	Mixed	PXFG	45/166	12/29/4	75/73/18	0.970	7
Gupta, 2014	India	West Asian	POAG	144/173	101/35/8	137/34/2	0.946	8
Gupta, 2014	India	West Asian	PCAG	87/173	73/14/0	137/34/2	0.946	8
Jünemann, 2005	Germany	Caucasian	POAG	76/71	32/37/7	45/24/2	0.569	7
Jünemann, 2005	Germany	Caucasian	PXFG	71/71	36/29/6	45/24/2	0.569	7
Mabuchi, 2006	Japan	East Asian	POAG	264/106	105/113/46	48/39/19	0.035	7
Micheal, 2009	Pakistan	West Asian	POAG	173/143	123/49/1	101/41/1	0.144	7
Micheal, 2009	Pakistan	West Asian	PCAG	122/143	84/26/12	101/41/1	0.144	7
Mossbock, 2006	Austria	Caucasian	POAG	204/211	119/71/14	105/86/20	0.696	7
Mossbock, 2006	Austria	Caucasian	PXFG	138/211	72/50/16	105/86/20	0.696	7
Nilforoushan, 2012	Iran	West Asian	POAG	73/90	39/28/6	53/33/4	0.688	8
Nilforoushan, 2012	Iran	West Asian	PXFG	85/90	46/31/8	53/33/4	0.688	8
Shi, 2013	China	East Asian	PCAG	231/306	81/106/44	93/152/61	0.938	8
Turaçli, 2005	Turkey	Caucasian	PXFG	76/34	39/31/6	18/12/4	0.382	7
Woo, 2009	Korea	East Asian	POAG	78/100	25/34/19	31/50/19	0.884	8
Zacharaki, 2014	Greece	Caucasian	POAG	64/130	22/31/11	39/70/21	0.264	8
Zacharaki, 2014	Greece	Caucasian	PXFG	72/130	29/33/10	39/70/21	0.264	8
Zetterberg, 2007	Sweden	Caucasian	POAG	243/187	126/97/20	89/75/23	0.252	7

Note

HWE, Hardy–Weinberg equilibrium; NA, Not available; NOS, Newcastle‐Ottawa scale; PCAG, Primary closed angle glaucoma; POAG, Primary open‐angle glaucoma; PXFG, Pseudoexfoliation glaucoma.

### 3.2 Overall and subgroup analyses

A total of 18 studies with 7,168 participants were analyzed. In overall analyses, a significant association with the likelihood of glaucoma was detected for the rs1801133 polymorphism in dominant (*p* = 0.04, OR = 0.90, 95%CI 0.81–1.00) and allele (*p* = 0.02, OR = 0.91, 95%CI 0.84–0.98) comparisons. Further subgroup analyses by ethnicity revealed that both rs1801131 and rs1801133 polymorphisms were significantly associated with the likelihood of glaucoma in West Asians. However, no positive results were detected for two investigated polymorphisms in East Asians and Caucasians. When we stratified data based on type of disease, we found that the rs1801133 polymorphism was also significantly associated with the likelihood of primary open‐angle glaucoma (POAG). No any other positive findings were observed in overall and subgroup analyses (see Table 2 and Data S1).

**Table 2:** Results of overall and subgroup analyses for *MTHFR* polymorphisms and glaucoma

Population	Sample size	Dominant comparison			Recessive comparison			Additive comparison			Allele comparison		
		*P* value	OR (95%CI)	*I* ^2^ statistic	*P* value	OR (95%CI)	*I* ^2^ statistic	*P* value	OR (95%CI)	*I* ^2^ statistic	*P* value	OR (95%CI)	*I* ^2^ statistic
rs1801131 A/C													
Overall	1178/995	0.71	0.95 (0.70–1.27)	19%	0.07	1.19 (0.98–1.44)	21%	0.13	0.87 (0.72–1.04)	0%	0.15	0.91 (0.79–1.04)	20%
East Asian	342/206	0.97	0.98 (0.24–3.96)	0%	0.50	1.14 (0.78–1.67)	0%	0.50	0.88 (0.60–1.29)	0%	0.55	0.91 (0.65–1.25)	0%
West Asian	295/286	**0.04**	**0.62 (0.39**–**0.98)**	0%	**0.003**	**1.91 (1.24**–**2.94)**	0%	0.36	0.85 (0.60–1.20)	0%	**0.007**	**0.72 (0.58**–**0.92)**	0%
Caucasian	383/453	0.09	1.51 (0.94–2.42)	0%	0.99	1.00 (0.75–1.32)	0%	0.32	0.87 (0.65–1.15)	0%	0.43	1.09 (0.88–1.35)	0%
POAG	824/669	0.80	1.05 (0.71–1.56)	0%	0.20	1.16 (0.93–1.46)	20%	0.16	0.86 (0.69–1.07)	0%	0.40	0.93 (0.79–1.10)	0%
PXFG	232/183	0.57	1.19 (0.65–2.18)	0%	0.68	0.92 (0.60–1.40)	0%	0.99	1.00 (0.65–1.55)	0%	0.55	1.10 (0.81–1.52)	15%
rs1801133 C/T													
Overall	3453/3715	**0.04**	**0.90 (0.81**–**1.00)**	43%	0.08	1.16 (0.98–1.38)	9%	0.30	1.06 (0.95–1.17)	28%	**0.02**	**0.91 (0.84**–**0.98)**	45%
East Asian	970/713	0.58	0.94 (0.76–1.16)	22%	0.74	1.05 (0.78–1.42)	0%	0.76	1.03 (0.84–1.27)	22%	0.57	0.96 (0.82–1.11)	0%
West Asian	894/1372	**0.02**	**0.80 (0.66**–**0.97)**	20%	**<0.001**	**3.07 (1.64**–**5.76)**	18%	0.29	1.11 (0.91–1.35)	36%	**0.002**	**0.77 (0.65**–**0.91)**	25%
Caucasian	1080/1148	0.40	0.88 (0.65–1.18)	61%	0.94	1.01 (0.76–1.34)	0%	0.99	1.00 (0.84–1.19)	32%	0.34	0.90 (0.71–1.13)	62%
POAG	2244/2019	**0.02**	**0.86 (0.76**–**0.98)**	50%	0.19	1.16 (0.93–1.46)	2%	0.13	1.10 (0.97–1.26)	33%	**0.04**	**0.85 (0.73**–**1.00)**	53%
PCAG	506/902	0.35	1.13 (0.88–1.45)	0%	0.54	1.87 (0.25–13.87)	74%	0.15	0.84 (0.65–1.07)	0%	0.76	1.03 (0.85–1.25)	34%
PXFG	703/794	0.16	0.85 (0.69–1.06)	44%	0.45	1.15 (0.80–1.64)	0%	0.34	1.11 (0.89–1.39)	31%	0.15	0.89 (0.75–1.05)	33%

Note

CI, Confidence interval; NA, Not available; OR, Odds ratio; POAG, Primary open‐angle glaucoma; PCAG, Primary closed angle glaucoma; PXFG, Pseudoexfoliation glaucoma.

*MTHFR*, NCBI Reference Sequence: NG_013351.1

The values in bold represent there are statistically significant differences between cases and controls.

### 3.3 Sensitivity analyses

Sensitivity analyses were conducted to examine the stability of synthetic results by eliminating studies that deviated from HWE. No changes of results were found in any comparisons, which indicated that our findings were statistically reliable.

### 3.4 Publication biases

Potential publication biases in the current study were evaluated with funnel plots. No obvious asymmetry of funnel plots was observed in any comparisons, which suggested that our findings were unlikely to be influenced by severe publication bias.

## 4 DISCUSSION

To the best of our knowledge, this is so far the most comprehensive meta‐analysis on correlations between *MTHFR* polymorphisms and glaucoma. The overall and subgroup analyses revealed that rs1801131 and rs1801133 polymorphisms were both significantly associated with the likelihood of glaucoma in West Asians. Nevertheless, no positive results were detected for two investigated polymorphisms in East Asians and Caucasians. The stability of synthetic results was subsequently evaluated in sensitivity analyses, and no changes of results were observed in any comparisons, which indicated that our findings were quite stable and reliable.

There are several points that need to be addressed about this meta‐analysis. Firstly, no obvious heterogeneities were detected in overall analyses for two investigated polymorphisms, which indicated that eligible studies could be considered as homogeneous, and thus synthesize the results of these studies is statistically feasible. Secondly, previous experimental studies have shown that the C to T substitution of rs1801133 (substitution of valine for alanine) and A to C substitution of rs1801131 (substitution of glutamate for alanine) could lead to reduced enzymatic activity and result in hyperhomocysteinemia, which may partially explain our positive findings (Li et al., 2017; Li, Dai, Zheng, Liu, & Huang, 2015). Thirdly, the pathogenic mechanism of glaucoma is quite complex, and hence it is unlikely that a single gene polymorphism can significantly contribute to its development. Therefore, to better illustrate potential correlations of certain gene polymorphisms with glaucoma, we strongly recommend further studies to perform haplotype analyses and explore potential gene–gene interactions.

As with all meta‐analysis, this study certainly has some limitations. First, our findings were based on unadjusted analyses due to lack of raw data, and failure to conduct further adjusted analyses for age, gender, and co‐morbidity conditions may impact the reliability of our findings (Shi, Xie, Jia, & Li, 2016; Xie, Shi, Xun, & Rao, 2017). Second, association between *MTHFR* polymorphisms and glaucoma may be affected by gene–gene and gene–environmental interactions. However, the majority of studies did not consider these potential interactions, which impeded us to perform relevant analyses accordingly (Xie, Shi, & Liu, 2017). Third, only retrospective case–control studies were included in this meta‐analysis, and thus direct causal relation between *MTHFR* polymorphisms and glaucoma could not be established (Zhao, Yin, Wang, & Si, 2015). Taken these limitations into consideration, the results of the current study should be interpreted with caution.

Overall, our meta‐analysis suggested that rs1801131 and rs1801133 polymorphisms may serve as genetic biomarkers of glaucoma in West Asians. However, further well‐designed studies are still warranted to confirm our findings.

## ETHICAL APPROVAL

This article does not contain any studies with human participants or animals performed by any of the authors.

## CONFLICT OF INTEREST

None declared.

## AUTHORS' CONTRIBUTIONS

Ling Zhang and Bin Chen conceived of the study, participated in its design, conducted the systematic literature review, performed data analyses, and drafted the manuscript. All authors have read and approved the final manuscript.
