# Supplementary material for: Correlation between MTHFR polymorphisms and glaucoma: A meta‐analysis
Source: Mol Genet Genomic Med. 2019 Mar 9;7(4):e00538. doi: 10.1002/mgg3.538 (PMC6465672; doi:10.1002/mgg3.538)
Supplement: Supplementary file 1 [file MGG3-7-na-s001.docx]

Forest plots of *MTHFR* polymorphisms and glaucoma


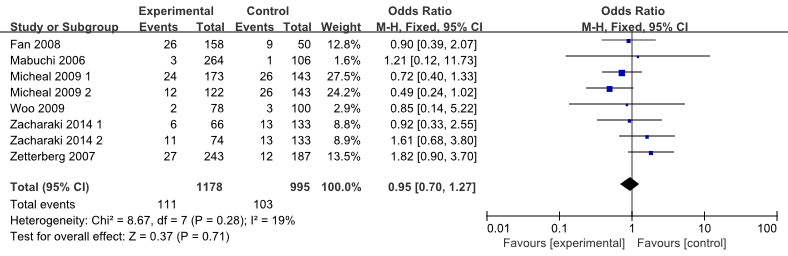


Forest plot of rs1801131 polymorphism and glaucoma under dominant comparison


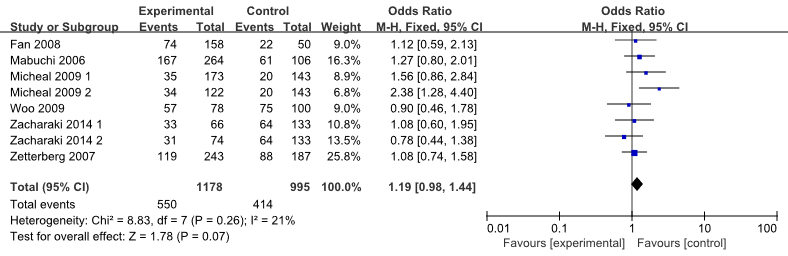


Forest plot of rs1801131 polymorphism and glaucoma under recessive comparison


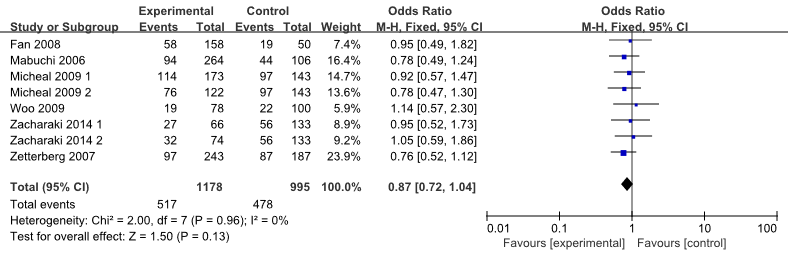


Forest plot of rs1801131 polymorphism and glaucoma under additive comparison


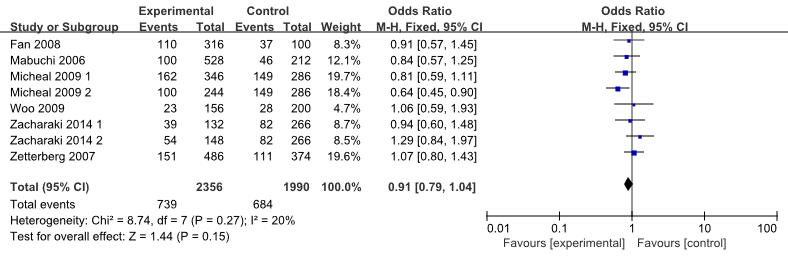


Forest plot of rs1801131 polymorphism and glaucoma under allele comparison


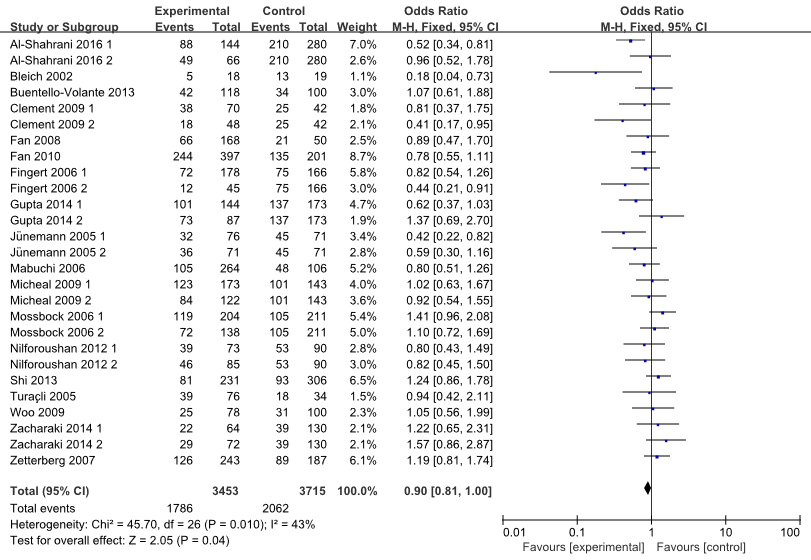


Forest plot of rs1801133 polymorphism and glaucoma under dominant comparison


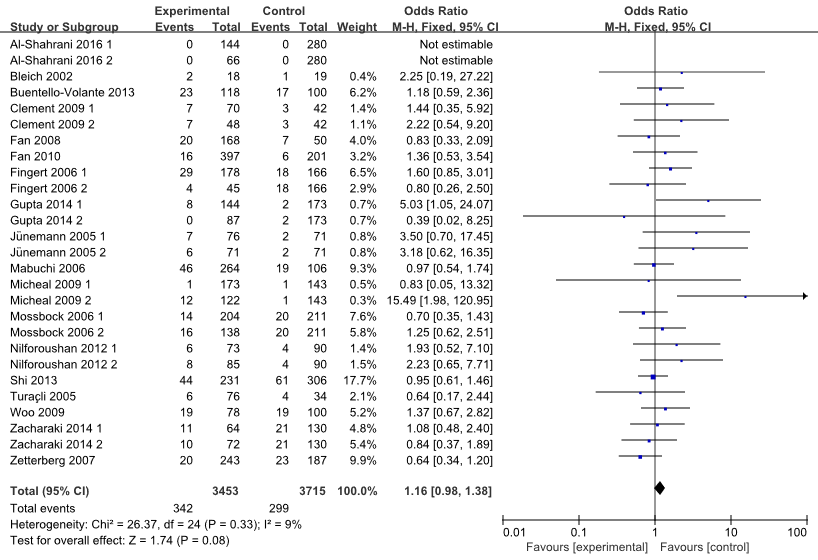


Forest plot of rs1801133 polymorphism and glaucoma under recessive comparison


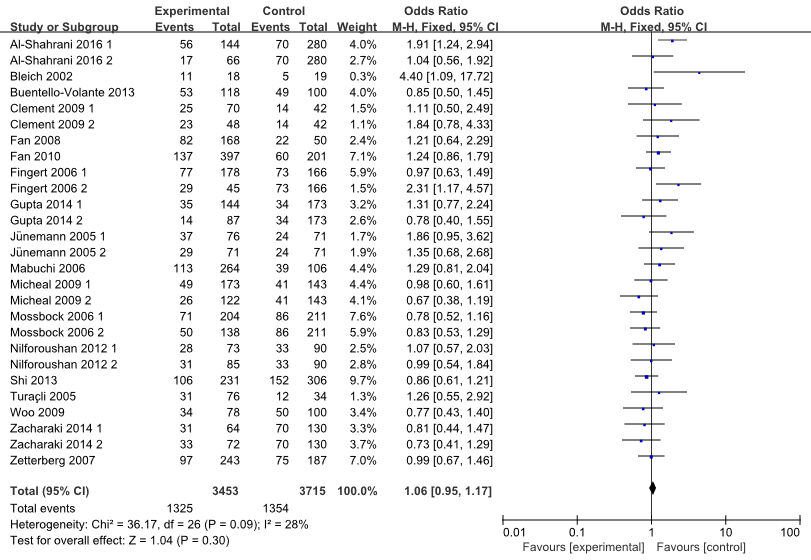


Forest plot of rs1801133 polymorphism and glaucoma under additive comparison


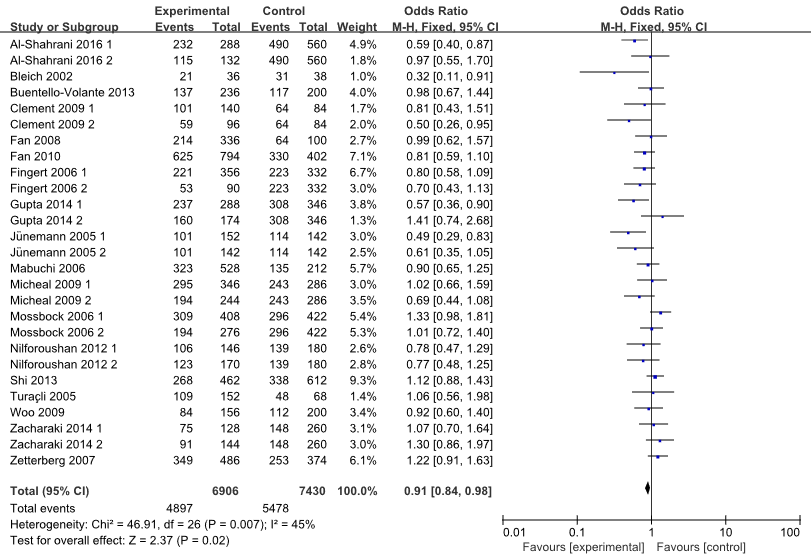


Forest plot of rs1801133 polymorphism and glaucoma under allele comparison
